# Zinc oxide nanoparticles as a substitute for zinc oxide or colistin sulfate: Effects on growth, serum enzymes, zinc deposition, intestinal morphology and epithelial barrier in weaned piglets

**DOI:** 10.1371/journal.pone.0181136

**Published:** 2017-07-13

**Authors:** Chao Wang, Ligen Zhang, Weipeng Su, Zhixiong Ying, Jintian He, Lili Zhang, Xiang Zhong, Tian Wang

**Affiliations:** College of Animal Science and Technology, Nanjing Agricultural University, Nanjing, People’s Republic of China; Institute of Materials Science, GERMANY

## Abstract

The objective of this study was to evaluate effects of zinc oxide nanoparticles (nano-ZnOs) as a substitute for colistin sulfate (CS) and/or zinc oxide (ZnO) on growth performance, serum enzymes, zinc deposition, intestinal morphology and epithelial barrier in weaned piglets. A total of 216 crossbred Duroc×(Landrace×Yorkshire) piglets weaned at 23 days were randomly assigned into 3 groups, which were fed with basal diets supplemented with 20 mg/kg CS (CS group), 20mg/kg CS+3000 mg/kg ZnO (CS+ZnO group), and 1200 mg/kg nano-ZnOs (nano-ZnO group) for 14 days. Results indicated that compared to CS group, supplementation of 1200 mg/kg nano-ZnOs (about 30 nm) significantly increased final body weight and average daily gain, and 3000 mg/kg ZnO plus colistin sulfate significantly increased average daily gain and decreased diarrhea rate in weaned piglets. There was no significant difference in growth performance and diarrhea rate between nano-ZnO and CS+ZnO groups. Supplementation of nano-ZnOs did not affect serum enzymes (glutamic oxalacetic transaminase, glutamic-pyruvic transaminase, and lactate dehydrogenase), but significantly increased plasma and tissue zinc concentrations (liver, tibia), improved intestinal morphology (increased duodenal and ileal villus length, crypt depth, and villus surface), enhanced mRNA expression of ZO-1 in ileal mucosa, and significantly decreased diamine oxidase activity in plasma, total aerobic bacterial population in MLN as compared to CS group. Effects of nano-ZnOs on serum enzymes, intestinal morphology, and mRNA expressions of tight junction were similar to those of high dietary ZnO plus colistin sulfate, while nano-ZnOs significantly reduced zinc concentrations of liver, tibia, and feces, and decreased total aerobic bacterial population in MLN as compared to CS+ZnO group. These results suggested that nano-ZnOs (1200 mg/kg) might be used as a substitute for colistin sulfate and high dietary ZnO in weaned piglets.

## Introduction

Weaning is commonly practiced at the 14^th^ to 28^th^ day of life in piglets for optimum herd performance. However, early weaning practice is closely associated with increased diarrhea occurrence, damaged epithelial barrier and restricted growth performance. Antibiotic growth promoters like colistin sulphate (CS) had been widely used as feed additive to attenuate gastrointestinal infections and improve the post-weaning growth performance [1–3]. But the use of sub-therapeutic antibiotics in feed is involved in antibiotic resistance potential of microbiota and residues in animal products. Due to these serious human health hazards, Europe has banned use of antibiotics as feed additives since January 2006 [4,5]. The other countries are trying to gradually reduce or forbid use of feed antibiotics. For example, the use of CS as feed additives in animal diets has been banned in China since May, 2017. Therefore, it is urgent to explore novel alternatives of antibiotic feed additives.

Zinc is an essential trace element for animals and serves as a component of many metalloenzymes, including DNA and RNA synthestases, and plays important roles in metabolism and intestinal nutrient absorption [6,7]. Physiological zinc requirement of nursery piglets is about 80–100 mg/kg [7,8], however, higher dietary doses of zinc oxide (ZnO) e.g 2000–4000 mg/kg are generally used to promote growth performance, reduce intestinal permeability and/or decrease incidence of diarrhea in weaning piglets [9–11]. Due to its low digestibility, most of dietary ZnO is excreted into the manure, which contains high amounts of zinc and may pose environmental pollution hazards [12,13].

Rapid developments in nanotechnology provide new dimensions for researches on substitutes of antibiotics and high dietary ZnO. Nanoparticles with the size between 1 to 100 nm (high surface area to volume ratio) exhibit quantum mechanics and show great potentials for applications in many fields [14–16]. In medicine, using nanoparticles as an alternative to traditional therapies shows various advantages, such as enhanced drug absorption, improved bioavailability and targeted activity for particular organs [17,18]. Nano-ZnOs are one of the best studied and most widely used nanoparticles for their high surface area, enhanced bioactivities, and especially for their high chemical stability and easy synthesis, therefore nano-ZnOs has yet been widely used in cosmetics, sunscreens, plastics and package [16,19–22]. It has also been well documented that nano-ZnOs shows great potentials as anti-cancer drugs and novel immunoprotective agents [23, 24].

Despite a few concerns raised about the toxicity of nano-ZnOs [25,26], it has been reported recently that nano-ZnOs still exhibit great promise in agriculture, such as feed additives [27–30]. As compared to their counterpart, nano-ZnOs exhibit enhanced antibacterial activities (comparable to colistin), especially against gram-negative bacteria [3,31,32]. Reddy et al. [33] also verified that nano-ZnOs possess strong antibacterial activity, exhibit low toxicity to eukaryotic systems [34]. Our previous studies have found that dietary supplementation of nano-ZnOs (500 mg/kg) for 32 weeks in mice showed minimum toxicity [35]. The oral zinc sulfate, as a common zinc source in animal diets, seems to be much more toxic than nano-ZnOs [36].

Based on these previous studies, we hypothesized that nano-ZnOs might be a potential substitute for CS and/or high dietary ZnO to improve growth performance, decrease diarrhea incidence, provide protection against intestinal injury and decrease fecal zinc content in weaned piglets. Therefore, this study was conducted to evaluate effects of nano-ZnOs on growth performance, enzymes, zinc deposition, intestinal morphology and epithelial barrier in weaned piglets. Results of present study might provide insights for application of nano-ZnOs as feed additive to replace feed antibiotics and/or high dietary ZnO.

## Material and methods

Experiments were approved and conducted according to the guidelines of Institutional Animal Care and Use Committee of Nanjing Agricultural University, China.

### Characteristics of nano-ZnOs

The morphological characteristics of nano-ZnOs were analyzed by the transmission electron microscope (TEM, JEM-200CX, Japan). The nano-ZnOs were provided by Zhangjiagang Bonded Area Hualu Nanometer Material Co., Ltd (China, Jiangsu). These nanoparticles were suspended in ethanol by ultrasonic vibration for 15 min. Subsequently, mixture was placed on a carbon coated copper grid and analyzed at 200 kv.

### Animal and experimental design

A total of 216 crossbred Duroc×(Landrace×Yorkshire) piglets weaned at 23 days of age were randomly allotted to 3 groups. Each group consisted of 72 piglets reared in 3 pens (24 piglets per pen representing one replicate). The piglets were fed with different diets as: (1) CS group: basal diet+20 mg/kg CS; (2) CS+ZnO group: basal diet+20 mg/kg CS+3000 mg/kg ZnO; (3) nano-ZnO group: basal diet+1200 mg/kg nano-ZnOs. Basal diet was formulated according to nutrient requirements of piglets [7], as shown in Table 1.

**Table 1:** Ingredients and chemical composition of basal diet (feed basis).

Ingredients	%	Composition^b^	
Corn	54.50	Digestible energy (Mcal/kg)	3.44
Soybean meal	18.00	Crude protein (%)	21.15
Soybean oil	2.50	Lysine (%)	1.42
Extruded soybean meal	10.00	Methionine (%)	0.43
Fish meal	4.50	Threonine (%)	0.86
Dried whey	5.00	Ca (%)	0.87
Glucose	2	P (%)	0.71
Dicalcium phosphate	0.80	Zn (mg/kg)	164.97
Limestone	0.90		
Sodium chloride	0.30		
Lysine	0.35		
Methionine	0.10		
Threonine	0.05		
Vitamin-mineral premix^a^^,^	1.0		

^a^ Supplied per kilogram diet as feed basis: Vitamin A, 5000 IU; Vitamin D3, 800 IU; Vitamin E, 30 IU; Vitamin K3, 1.0 mg; Biotin, 0.05mg; Folic acid, 0.3mg; Niacin, 10 mg; D-pantothenic acid, 10mg; Riboflavin, 3.6mg; Thiamine, 1.0mg; Pyridoxin, 1.5mg; Choline, 800mg; Zn (ZnSO_4_):120mg; Fe (FeSO_4_), 125mg; Cu (CuSO_4_·5H_2_O),15mg/kg; Mn (MnSO_4_·H_2_O), 10mg/kg; I (KI), 0.15 mg; Se (Na_2_SeO_3_), 0.2 mg; enramycin, 20 mg; chlortetracycline, 50 mg.

^b^For the composition, digestible energy, lysine, methionine and threonine were calculated, while others were analyzed.

The piglets were obtained and raised in a local farm. During this 14 day trial, piglets were housed in a warm (20–28°C) house with concrete floors (4.2×5.0 m per pen). A nipple drinker and feeders with wide trough were provided to allow pigs ad libitum access to water and feed. House temperature and ventilation system, food and water supply, and swine behavior patterns (patterns of sleeping, walking, breathing, feeding and drinking) were monitored three times a day (7:00, 14:00 and 21:00). Body weight (BW), feed intake and incidence of diarrhea were recorded during this 14 day trial. The average daily gain (ADG), average feed intake (ADFI), feed/gain ratio (F/G) and diarrhea rate were calculated. After feeding trial, two piglets (one male and one female, fasting for 6h) from each replicate were randomly selected, euthanized by electrical stunning and exsanguinated. Plasma and serum were collected by centrifugation at 3000×g for 15 min in tubes with or without heparin sodium, and stored at -80°C. The samples of tibia, liver and feces were stored at -20°C to determine zinc concentration.

### Analysis of serum and plasma parameters

The serum activities of glutamic oxaloacetic transaminase (GOT), glutamic-pyruvic transaminase (GPT) and lactate dehydrogenase (LDH) were analyzed by corresponding commercial kits provided by Nanjing Jiancheng Bioengineering Institute (Nanjing, China). The D-lactic acid content, diamine oxidase (DAO) activity and endotoxin level in plasma were determined by ELISA kits (Nanjing Aoqing Co., Ltd, Jiangsu, China).

### Analysis of zinc concentrations in plasma, tibia, liver and feces

The zinc concentrations in plasma, tibia, liver and feces were determined as previously reported with minor modifications [37]. Briefly, plasma samples were diluted with demineralized water. Samples of tibia, liver and feces (1–2.5g) were digested with the acid mixture (4:1 HNO_3_ and HClO_4_). The digest was brought to a volume of 50 ml with demineralized water and diluted to the optimal concentration with 5% HNO_3_ solution. After the external matrix-matched standard curves were prepared via zinc standard (diluted with 5% HNO_3_ solution), blanks and prepared samples were determined by inductively coupled plasma optical emission spectrometry (ICP-OES).

### Analysis of the population of total aerobic bacteria

After the mesenteric lymph node (MLN) was collected from the ileum, they were packed in sterile plastic tube and were homogenized with phosphate buffer solution (PBS) on ice (1:9 w/v). The homogenate was used as a source for serial dilutions in PBS for viable counts of total aerobic bacteria. Subsequently, 100 μl of serial dilution was planted in nutrient agar plate, which was cultured at 37°C for 24h. The bacterial enumerations were expressed as log(cfu/g).

### Analysis of intestinal morphology

The intestinal morphology was analyzed as previously described by Dong et al. [38] with minor modifications. Briefly, 2 cm-long segments of duodenum (about 4 cm from pyloric sphincter) and ileum (about 15 cm beyond ileocecal junction) were harvested, fixed in paraformaldehyde, dehydrated using a graded series of ethanol and embedded in paraffin. Cross sections (5 microns in size) were cut, dehydrated, stained with hematoxylin and eosin (HE). For each section, villus length, crypt depth and villus width were determined with an optical binocular microscope (Olympus BX5, Olympus Optical Co. Ltd, Japan) and Image-Pro Plus software. The villi/crypt ratio and villus area were calculated.

### Analysis of relative mRNA expression of occludin, claudin-2 and ZO-1

Samples of ileal mucosa were scraped on ice, frozen in liquid nitrogen immediately and stored at -80°C. Total RNA was extracted with Trizol reagents according to manufacturer’s instruction (Invitrogen, USA). RNA was quantified by nano-drop 2000 (absorbance ratios of 260/280 nm and 260/230 nm between 1.90 and 2.05) and verified by agarose gel electrophoresis. Then cDNA was prepared with Primer-Script^TM^ reagent kit, provided by TakaRa Biotechnology Co. Ltd (Dalian, China).

The gene-specific primers of occludin, claudin-2 and ZO-1 were synthesized by Invitrogen Biotech Co. Ltd (Shanghai, China) and are listed in Table 2. GAPDH was chosen as a housekeeping gene. Reverse transcription polymerase chain reaction (RT-PCR) tests were conducted with ABI 7600 RT-PCR system with a SYBR Premix Ex Taq^TM^ kits (TakaRa Biotechnology Co. Ltd Dalian, China). The relative mRNA expression was analyzed with ABI software and calculated with 2^-ΔΔCt^ as described previously [39].

**Table 2:** Primer sequences used in quantitative real time PCR assays.

Gene	Accession No.	Sequence (5’ to 3’)	Size (bp)
Claudin-2	NM_001161638.1	F-ACTGCAAGGAAATCGCTCCA	98
		R-TCTTGGCTTTGGGTGGTTGA	
Occludin	NM_001163647.2	F-CAGGTGCACCCTCCAGATTG	75
		R-CAGCGGGTCACCTGATCTTC	
ZO-1	XM_005659811.1	F-GACCCGGCCAAGGTGTATAG	75
		R-TGGCTGCTTCAAGACATGGT	
GAPDH	NM_001206359.1	F-CGTCCCTGAGACACGATGGT	98
		R-GCCTTGACTGTGCCGTGGAAT	

### Statistical analysis

All data were analyzed by the SPSS statistical package (IBM SPSS, version 20.0, Chicago) and expressed as mean ± standard error (SE). The statistical analysis was performed using Analysis of Variance (ANOVA) with comparison of means by Duncan’s Multiple Comparison Test. Pens were used as experimental units for the analysis of growth performance, diarrhea rate and zinc concentration in feces. For other parameters, individual piglet was used as the experimental unit. P value less than 0.05 was considered as significant difference, while a P value between 0.05 and 0.10 was considered as a tendency towards statistical difference.

## Results

### Characteristics of nano-ZnOs

**Fig 1:**
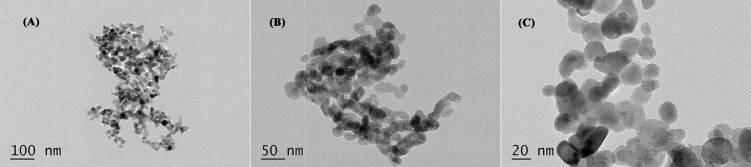
TEM images of nano-ZnOs with low (A), middle (B) and high (C) magnification.

The characteristics of nano-ZnOs were analyzed by TEM, and images with low, middle and high magnification were shown in Fig 1. Results indicated that primary particle sizes were about 30 nm (mainly range from 20 to 40 nm) and these nano-ZnOs exhibited almost spherical geometry.

### Effects of nano-ZnOs on growth performance and diarrhea rate

Effects of nano-ZnOs on growth performance and diarrhea rate are presented in Table 3. Dietary supplementation of nano-ZnOs significantly increased final BW and ADG (P<0.05), while had no significant effects on ADFI, and F/G (P>0.05). There was no significant difference in growth performance and diarrhea rate between nano-ZnO group and CS+ZnO group (P>0.05), although piglets in CS+ZnO group had significantly lower diarrhea rate than those in CS group (P<0.05).

**Table 3:** Effect of dietary nano-ZnOs on growth performance and diarrhea ratio in weaned piglets.

Item^1^	Treatments^2^			*P*
	CS	CS+ZnO	Nano-ZnO	
Initial BW (kg)	8.09±0.11	7.82±0.18	8.10±0.17	0.42
Final BW (kg)	10.68±0.13^b^	11.11±0.05^a^^b^	11.35±0.19^a^	0.03
ADG (g)	185.09±0.93^b^	235.01±15.97^a^	232.54±9.21^a^	0.03
ADFI (g)	263.98±10.80	317.88±19.31	293.97±9.29	0.09
F:G	1.43±0.05	1.36±0.04	1.27±0.02	0.07
Diarrhea rate	9.31±1.63^a^	3.84±0.74^b^	5.70±0.74^a^^b^	0.04

^a-b^ Means in a row with different superscripts were significantly different (*P*<0.05).

^1^Data were expressed as mean ± SE (n = 3).

^2^Treatments including: CS group: basal diet+20 mg/kg CS; CS+ZnO group: basal diet+20 mg/kg CS+3000 mg/kg ZnO;nano-ZnO group: basal diet+ 1200 mg/kg nano-ZnOs.

### Effects of nano-ZnOs on serum and plasma enzymes

As shown in Table 4, dietary supplementation of nano-ZnOs and CS+ZnO did not affect activities of serum GOT, GPT, and LDH (P>0.05) but significantly decreased DAO activity in plasma as compared with CS group (P<0.01). Piglets in nano-ZnO and CS+ZnO group showed a tendency to have lower levels of D-lactic acid (P = 0.06) and endotoxin (P = 0.05) in plasma as compared with CS group.

**Table 4:** Effect of dietary nano-ZnOs on D-lactic acid, endotoxin and DAO in plasma.

Item^1^	Treatments^2^			*P*
	CS	CS+ZnO	Nano-ZnO	
**Serum**				
LDH(U/L)	337.45±19.35	336.64±20.08	347.76±14.30	0.89
GOT(U/L)	26.44±2.97	26.75±1.12	33.83±4.47	0.21
GPT(U/L)	22.37±2.28	19.96±2.04	20.62±1.47	0.67
**Plasma**				
D-lactic acid (μg/L)	516.28±14.43	466.27±25.67	449.86±13.80	0.06
Endotoxin(EU/L)	0.42±0.01	0.38±0.01	0.34±0.03	0.05
DAO(U/ml)	11.57±0.49^a^	9.17±0.19^b^	8.71±0.59^b^	<0.01

^a-b^ Means in a row with different superscripts were significantly different (*P*<0.05).

^1^Data were expressed as mean ± SE (n = 6).

^2^Treatments including: CS group: basal diet+20 mg/kg CS; CS+ZnO group: basal diet+20 mg/kg CS+3000 mg/kg ZnO;nano-ZnO group: basal diet+ 1200 mg/kg nano-ZnOs.

### Effects of nano-ZnOs on zinc concentrations in plasma, liver, tibia and feces

Dietary supplementation of nano-ZnOs and CS+ZnO significantly enhanced zinc concentrations of plasma, liver, tibia and feces (P<0.001) in weaned piglets (Table 5). However, piglets from nano-ZnO group showed significantly lower zinc concentrations in liver, tibia and feces than those from CS+ZnO group (P<0.001).

**Table 5:** Effect of dietary nano-ZnOs on zinc concentration in plasma, liver, tibia and feces.

Item^1^		Treatments^2^		*P*
	CS	CS+ZnO	Nano-ZnO	
Plasma (mg/L)	1.56±0.09^b^	3.43±0.18^a^	3.37±0.19^a^	<0.001
Tibia (mg/kg)	138.77±6.37^c^	272.99±17.19^a^	212.04±12.90^b^	<0.001
Liver (mg/kg)	103.93±9.15^c^	884.96±24.69^a^	437.03±39.93^b^	<0.001
Feces (g/kg)	4.61±0.27^c^	12.57±0.35^a^	7.95±0.24^b^	<0.001

^a-b^ Means in a row with different superscripts were significantly different (*P*<0.05).

^1^Data were expressed as mean ± SE. For zinc concentrations of tibia and liver, n = 6; For feces, n = 3.

^2^Treatments including: CS group: basal diet+20 mg/kg CS; CS+ZnO group: basal diet+20 mg/kg CS+3000 mg/kg ZnO;nano-ZnO group: basal diet+ 1200 mg/kg nano-ZnOs.

### Effects of nano-ZnOs on intestinal morphology

**Fig 2:**
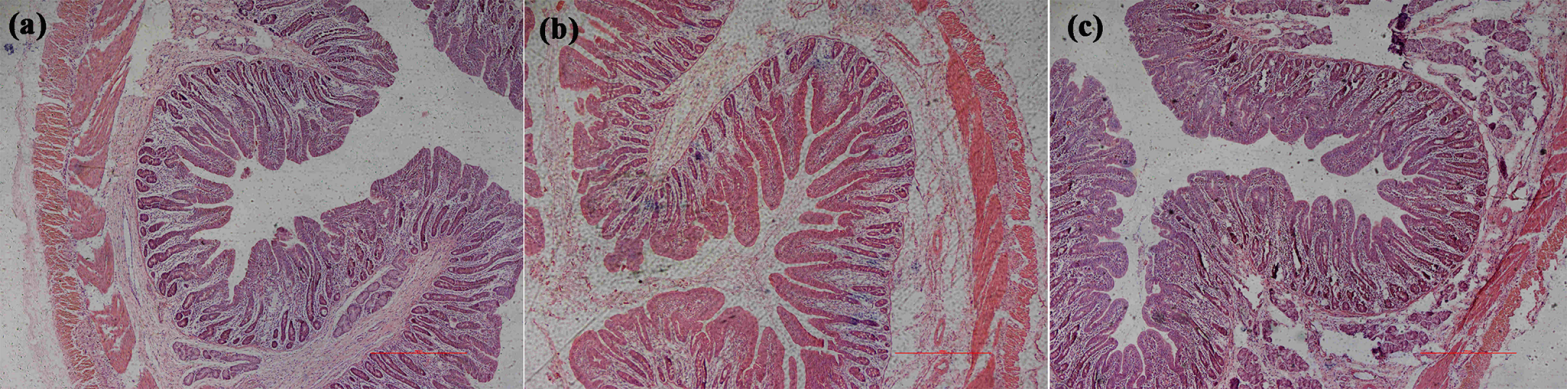
Images of duodenal morphology in weaned piglets from CS (a), CS+ZnO (b) and nano-ZnO (c) groups.

**Fig 3:**
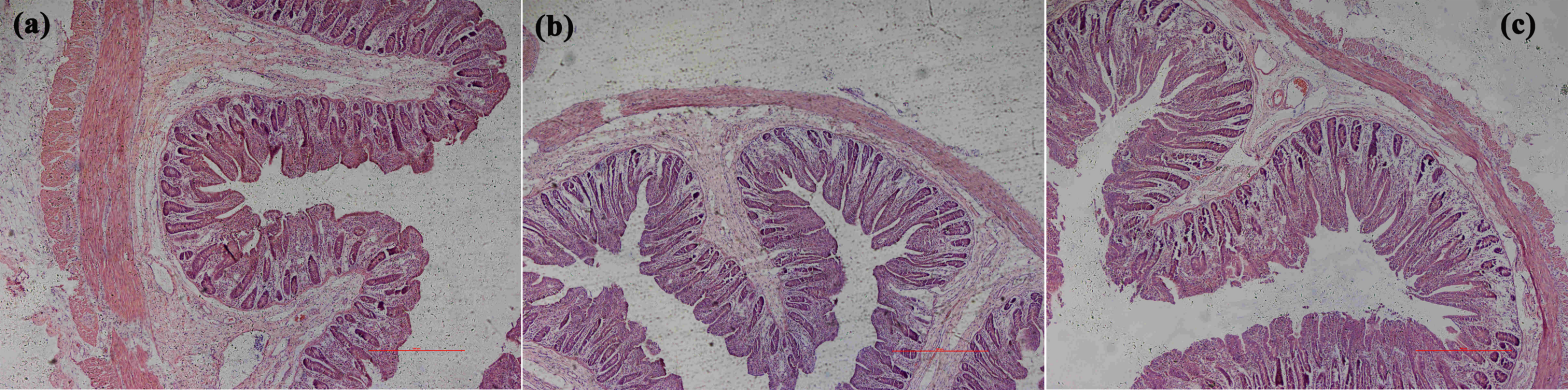
Images of ileal morphology in weaned piglets from CS (a), CS+ZnO (b) and nano-ZnO (c) groups.

Effects of nano-ZnOs on duodenal (Fig 2) and ileal (Fig 3) morphology were shown in Table 6. Results revealed that dietary nano-ZnOs and CS+ZnO did not affect duodenal villus width (P>0.05), but significantly increased duodenal villus length and villus surface area (P<0.05). Compared to CS group, supplementation of nano-ZnOs significantly increased crypt depth (P<0.01), while CS+ZnO significantly increased villi/crypt ratio (P<0.05). Analysis of ileal mucosal morphology revealed that dietary nano-ZnOs and CS+ZnO significantly increased villus length, crypt depth, villus width and villus surface area (P<0.05), while had no effects on villi/crypt ratio (P>0.05). There was no significant difference in ileal mucosal morphology between nano-ZnO and CS+ZnO groups (P>0.05).

**Table 6:** Effect of dietary nano-ZnOs on intestinal morphology.

Item^1^	Treatments^2^			*P*
	CS	CS+ZnO	Nano-ZnO	
**Duodenum**				
Villus length (μm)	279.68±4.48^b^	307.72±4.70^a^	317.36±7.85^a^	0.001
Crypt depth (μm)	238.65±1.78^b^	247.43±4.19^b^	273.98±5.96^a^	<0.001
Villi/crypt ratio	1.17±0.02^b^	1.25±0.03^a^	1.16±0.01^b^	0.02
Villus width (μm)	106.33±1.83	114.33±2.45	112.97±5.37	0.27
Villus surface area (mm^2^)	0.048±0.001^b^	0.056±0.002^a^	0.058±0.004^a^	0.046
**Ileum**				
Villus length (μm)	212.86±12.03^b^	240.19±3.86^a^	251.99±7.46^a^	0.02
Crypt depth (μm)	185.18±8.07^b^	206.58±3.85^a^	212.56±6.05^a^	0.02
Villi/crypt ratio	1.15±0.03	1.16±0.02	1.19±0.01	0.46
Villus width (μm)	79.97±3.03^b^	97.93±1.80^a^	101.45±2.13^a^	<0.01
Villus surface area (mm^2^)	0.027±0.002^b^	0.038±0.001^a^	0.041±0.002^a^	<0.01

^a-b^ Means in a row with different superscripts were significantly different (*P*<0.05).

^1^Data were expressed as mean ± SE (n = 6).

^2^Treatments including: CS group: basal diet+20 mg/kg CS; CS+ZnO group: basal diet+20 mg/kg CS+3000 mg/kg ZnO;nano-ZnO group: basal diet+ 1200 mg/kg nano-ZnOs.

### Effects of nano-ZnOs on total aerobic bacteria in MLN and mRNA expression of occludin, claudin-2, and ZO-1 in ileal mucosa

**Fig 4:**
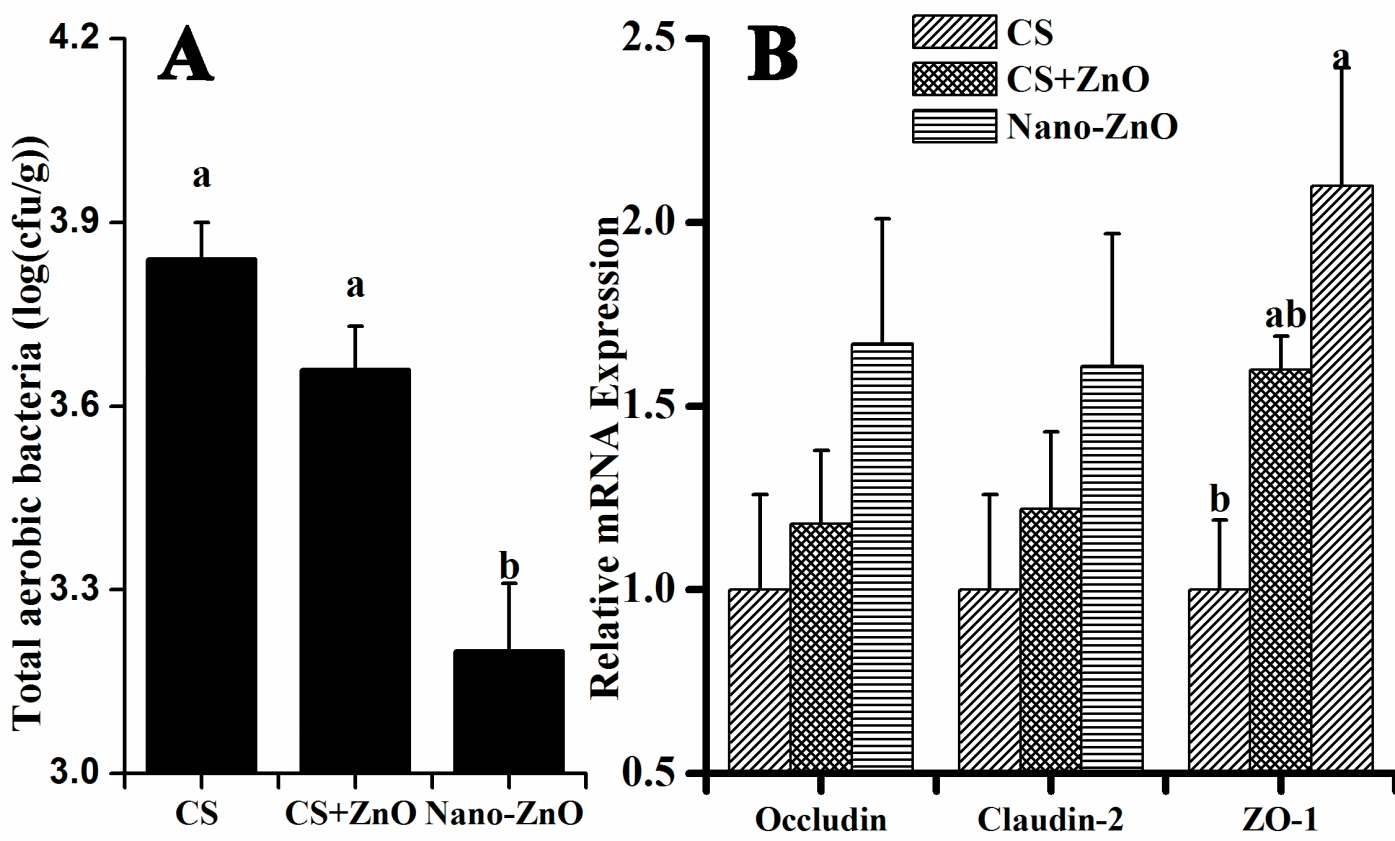
**Effects of nano-ZnOs on total aerobic bacteria in MLN (A) and mRNA expressions of occludin, claudin-2, and ZO-1 in ileal mucosal (B).** Data are expressed as mean± SE (n = 6). For the mRNA expression, data are normalized to the CS group. ^a-b^Means for the same parameter with different superscripts are significantly different (P<0.05).

Effects of nano-ZnOs on total aerobic bacteria in MLN and mRNA expression of occluding, claudin-2, and ZO-1 in ileal mucosa were shown in Fig 4. As shown in Fig 4A, dietary nano-ZnOs significantly decreased population of total aerobic bacteria in MLN as compared to CS and CS+ZnO group (P<0.05). Fig 4B showed that dietary supplemented nano-ZnOs and CS+ZnO did not affect the mRNA expression of occludin and claudin-2 (P>0.05), however, nano-ZnOs significantly increased expression of ZO-1 as compared to CS group (P<0.05). There was no significant difference in mRNA expressions between nano-ZnO and CS+ZnO groups (P>0.05).

## Discussion

Dietary supplementation of antibiotic growth promoters like CS and high dietary ZnO, is commonly practiced in piglets to address post-weaning challenges of reduced growth and high incidence of diarrhea [40–42]. Results of our present study showed that supplementation of dietary ZnO (3000 mg/kg) plus colistin sulfate (20 mg/kg) improved growth performance and decreased occurrence of diarrhea as compared to CS group, which is in line with previous reports [9,11].

In this study, effects of dietary 1200 mg/kg nano-ZnOs on growth performance and diarrhea ratio were similar to the beneficial effects of 3000 mg/kg ZnO plus colistin sulfate. Hahn and Baker [43] proposed that the mechanism for enhanced growth performance in weaned piglets was linked with plasma zinc concentration. Results of our study showed that dietary supplementation of 1200 mg/kg nano-ZnOs and 3000 mg/kg ZnO plus colistin sulfate increased plasma zinc concentration, and there was no significant difference in plasma zinc content between these two groups. Similarly, it has been reported earlier that nanoparticles exhibit higher bioavailability, and enhance drug absorption [29,44]. Early reports also verified that dietary supplementation of 20 and 60 mg/kg nano-ZnOs had greater weight gains and better feed conversion ratios than 60 mg/kg ZnO in broilers [28].

Nanoparticles are natural or artificial polymers with sizes ranging from 1 to 100 nm and have a larger surface area, which can facilitate more catalytic space, can easily enter and target cells, enhance antibacterial activities, and possess various potentials in applications [14,17,19]. The particle size and shape are important characteristics of nanoparticles, which are closely associated with their properties [45]. The antibacterial activity of nano-ZnOs increases with decrease in crystallite size [31,46]. Higher photocatalytic inactivation against E. coli had been observed for flower shaped particles than rod and sphere-like particles [46]. The characteristics of nano-ZnOs were determined by TEM in this study. The results revealed that these particles possessed potentials of nanoparticles since primary particle sizes were about 30 nm (mainly ranging from 20 to 40 nm). These nano-ZnOs were of nearly spherical geometry, similar to those used in our previous experiments on mice [35,36].

The toxicity of high doses of nano-ZnOs have been reported by several researchers [25,26,47,48]. Serum GOT, GPT and LDH activities are important biological parameters to evaluate the possible toxicity of nano-ZnOs in vivo. However, serum enzyme activities (GOT, GPT and LDH) were not affected by supplemented 1200 mg/kg nano-ZnOs or 3000 mg/kg ZnO plus colistin sulfate as compared to CS group in the present study, suggesting that dietary addition of nano-ZnOs and 3000 mg/kg ZnO plus colistin sulfate for 14 days produced minimal toxicity to weaned piglets. Similar to our findings, Wang et al. [35] also found that dietary supplementation of nano-ZnOs (500 mg/kg) for 32 weeks did not affect serum GOT and GPT activities in mice. However, supplementation of 5000 mg/kg nano-ZnOs for 32 weeks enhanced serum GPT activity in mice [35]. Wang et al. [48] reported that oral administration with high doses of 120-nm ZnO (1-5g/kg body weight) led to liver damage and increased serum LDH activity in mice. Our previous study also indicated that oral administration with 250 mg/kg nano-ZnOs and zinc sulfate for 7 weeks can cause liver damage, however, nano-ZnOs seemed to be safer than zinc sulfate [36]. More studies are required to explore long-term effects of nano-ZnOs on pigs and further investigate possible mechanisms in detail.

It has been reported that tissue damage induced by oral nano-ZnOs and zinc salt is closely related to zinc accumulation, which might further induce oxidative stress and DNA damage [25,26,36]. The analysis of zinc deposition in our present study showed that dietary nano-ZnOs (1200 mg/kg) and ZnO (3000 mg/kg) plus colistin sulphate increased zinc concentrations in liver and tibia as compared to CS group. However, zinc concentrations of liver and tibia in weaned piglets from nano-ZnO group were much lower than those of piglets in CS+ZnO group, suggesting that nano-ZnOs might be safer option than ZnO plus colistin sulfate. In addition, significantly lower level of fecal zinc content in nano-ZnO group revealed that use of nanoparticles may alleviate environmental pollution caused by high dietary ZnO [12,13,49].

Villus length, crypt depth, villi/crypt ratios, villus width, and villus surface area are important indicators of intestinal morphology, which play critical roles in nutrient absorption. In the present study, dietary supplementation of 1200 mg/kg nano-ZnOs was as efficacious as 3000 mg/kg ZnO plus colistin sulfate to increase villus width (in ileum), villus length (in both duodenum and ileum) and villus surface area (in both duodenum and ileum), which would be beneficial for intestinal nutrient absorption. The mucosal surfaces of small intestine are lined by epithelial cells, which are derived from self-renewing stem cells that reside at the crypt base [50,51]. In our present study, dietary addition of nano-ZnOs increased crypt depth in duodenum and ileum, which suggests that ability of intestinal stem cells for self-regeneration and proliferation might have been enhanced but it needs further investigations.

D-lactic acid, DAO, and endotoxin in plasma are important indicators for intestinal epithelial integrity and permeability. The impaired intestinal mucosal integrity may enhance activity of DAO, and increase contents of D-lactic acid and endotoxin [52,53]. Results of our present study revealed that nano-ZnOs and ZnO plus colistin sulfate significantly decreased plasma DAO activity and also tended to decrease contents of D-lactic acid and endotoxin as compared with CS group. These findings reinforced that nano-ZnOs and ZnO plus colistin sulfate showed protective effects on intestinal mucosal integrity.

To further investigate beneficial effects of nano-ZnOs on intestinal barrier, population of total aerobic bacteria in MLN was determined as it was reported that once intestinal epithelial barrier is impaired, bacterial translocation from gastrointestinal tract to MLN enhances [54]. Our present study revealed that dietary supplementation of nano-ZnOs decreased population of total aerobic bacteria in MLN as compared to CS group, which were in agreement with the decreased DAO activity in plasma. To further elucidate the related molecular mechanism, mRNA expressions of occludin, claudin-2 and ZO-1 in ileal mucosa were determined. The intestinal epithelial barrier is mainly modulated by tight junction, which is comprised of several proteins, such as occludin, claudin, and ZO-1 [53,55]. This junction combines with epithelial cells to maintain intestinal integrity and is an important indicator for intestinal barrier function [53,55]. In this study, dietary supplementation of nano-ZnOs increased mRNA expression of ZO-1 in ileal mucosa as compared to CS group, suggesting that dietary nano-ZnOs enhanced the expression of ZO-1 at mRNA level, which could decrease intestinal permeability and reduce intestinal infections [9,56]. There was no significant difference in mRNA expressions of occludin, claudin-2 and ZO-1 in ileal mucosal between nano-ZnO and ZnO plus colistin sulfate. These results suggested that the protective effect of nano-ZnOs on epithelial barrier was similar or even better than that of ZnO plus colistin sulfate, since total aerobic bacterial population in MLN was significantly decreased by nano-ZnOs as compared to CS+ZnO group.

## Conclusion

Dietary supplementation of nano-ZnOs (1200 mg/kg) was as efficacious as ZnO (3000 mg/kg) plus colistin sulphate (20 mg/kg) in promoting growth performance, alleviating diarrhea and increasing plasma zinc concentration. However, nano-ZnOs might be safer (decreased tissue zinc concentration), more beneficial for the environment (decreased fecal zinc concentration), and even seemed to be more effective on the epithelial barrier (decreased total aerobe bacterial population in MLN) than ZnO plus colistin sulfate. Therefore, nano-ZnOs could be used as a substitute for high dietary ZnO and colistin sulfate. Our results might be useful for future investigations on nano-ZnOs as feed additives and their application in agriculture. However, further investigations are required to explore long term effects of nano-ZnOs in piglets and in environmental organisms.
